# The Importance of Activating Factors in Physical Activity Interventions for Older Adults Using Information and Communication Technologies: Systematic Review

**DOI:** 10.2196/42968

**Published:** 2023-10-24

**Authors:** Ellen Bentlage, John Jnr Nyamadi, Rosemary Dubbeldam

**Affiliations:** 1 Department of Movement Science, Institute of Sport and Exercise Sciences, University of Münster, Münster, Germany

**Keywords:** older adults, information and communication technology, healthy aging, activation factors, skills, knowledge, motivation, behavior change techniques, physical activity

## Abstract

**Background:**

In an aging population, it is important to activate older adults in taking care of their own health. Increasing physical activity is one way to avoid or lessen age-related physical and mental impairments. Interest in the use of information and communication technology (ICT) tools to promote physical activity among older adults is growing considerably. Such tools are suitable for communicating activation factors—skills, knowledge, and motivation—by integrating a variety of behavior change techniques (BCTs) to enhance physical activity. Although activation factors have been incorporated into physical activity interventions using ICT, little is known about the actual integration methods used in such interventions or about the effects of activation factors on influencing behavior change.

**Objective:**

The first aim of this study was to identify which of the activation factors were covered in physical activity–promoting ICT interventions for older adults and which BCTs were used to address them. The second objective was to classify the user interaction interfaces and delivery modes that were used to promote these activation factors.

**Methods:**

The search engines of PubMed, Web of Science, and ScienceDirect were used to search for and identify articles examining the effectiveness of ICT interventions for promoting physical activity in older adults. References and related data were selected, extracted, and reviewed independently by 2 reviewers. The risk of bias was assessed, and any conflict was addressed by a third separate reviewer. Selected articles included older adults aged ≥55 years without pre-existing medical diseases and other physical or mental conditions that could hinder movement.

**Results:**

In total, 368 records were retrieved, and 13 studies met all inclusion criteria. Articles differed in terms of themes, timescales, user interaction interfaces, and outcome measures; therefore, a quantitative data synthesis was not feasible. Motivation was the most promoted activation factor among all trials (33 times). An app and a smartwatch were used in the majority of intervention groups (7/20, 35%) for tracking physical activity and receiving personalized feedback based on the individual goals. Skills (25 times) and knowledge (17 times) were the next most commonly addressed activation factors. Face-to-face interaction was the most used approach to targeting users’ skills, including providing instructions on how to perform a behavior and exchanging knowledge via education on the health consequences of insufficient physical activity. Overall, integrating all 3 activation factors and using multiple user interaction interfaces with a variety of delivery modes proved the most effective in improving physical activity.

**Conclusions:**

This study highlights commonly used BCTs and preferred modes of their delivery. So far, only a limited number of available BCTs (21/102, 21%) have been integrated. Considering their effectiveness, a larger variety of BCTs that address skills, knowledge, and motivation should be exploited in future ICT interventions.

## Introduction

### Consequences of Aging

The World Health Organization projects a 34% increase in the global population of 1 billion older adults by 2030, showing a demographic trend toward an older population [1]. Several pathologies, such as pulmonary disease, neurodegenerative disorders, and cardiovascular disease, share aging as their dominant pathogenesis risk factor, though these pathologies can be positively influenced by physical activity [2-4]. Hence, managing the health of the older population is important [5].

### Activation in Health Care Management

Individuals themselves can successfully be activated in their health management if they are equipped with activating factors. Activation of the individual leads to behavioral changes and consequently enhances, for example, physical activity levels [6]. Activating people involves making the individuals believe in their participatory role and fostering their confidence through improving their skills and abilities regarding their well-being. It also involves creating awareness by communicating knowledge on the necessity to act. Furthermore, it includes motivating individuals to take action to maintain and improve their health outcomes [7,8]. Evidence proves that activated individuals are independently able to better control their health and have better health outcomes [9]. As a result, activated individuals develop confidence in self–health care management [10].

### Activation Using Information and Communication Technology

First, information and communication technology (ICT) interfaces communicate activation factors effectively to the user. Such activation factors can be described by using the behavior change technique (BCT) taxonomy from Michie et al [11]. This taxonomy includes 16 categories for promoting skills, knowledge, or motivation; these categories include shaping knowledge, comparing behavior, natural consequences, comparison of outcomes, goals and planning, and feedback and monitoring. Further, considering the possibilities of ICT tools, Dugas and colleagues [12] added 2 more categories (ie, personalization and gamification), including 9 BCTs in total. A review by Aldawood et al [13] also pointed out that ICT interfaces in health interventions offer various BCTs that indeed raise awareness of health and promote more self-awareness among people. Second, ICT interventions that include activation factors are effective for improving health. Such interventions include, among others, remote coaching or monitoring, automated feedback, and increased accessibility to credible health information [14,15]. Providing feedback based on detected behavior patterns, when paired with reminders to be active, leads to improvements in physical activity behavior, better health-related knowledge, and increased motivation [16]. Additionally, a web-based intervention [17] and an intervention using wearable activity trackers connected with a smartphone app [18] both showed improvements in participants’ health skills, knowledge, and motivation for developing and maintaining positive health-related practices. Lastly, entertainment, such as exergames, can be used to teach health-related skills, provide feedback, and constantly motivate the user [19].

Although activation factors have been incorporated into physical activity interventions using ICT, little is known about the actual integration methods used in such interventions or about the effects of activation factors on influencing behavior change. McGarrigle and Todd [20] stated that ICT interventions incorporating BCTs may be more effective in promoting physical activity than those interventions that do not focus on such techniques. Accordingly, Dugas and colleagues [12] performed a systematic review on health behavior interventions within mobile health apps and reported on the integrated BCTs and how they influenced outcomes.

### Study Aims

This systematic review, as a primary objective, aimed to identify which of the activation factors—skills, knowledge, and motivation—were covered in ICT interventions that promote the physical activity of older adults and to report the incorporated BCTs. The secondary objective was to classify the user interaction interfaces and delivery modes that were used to promote the activation factors.

## Methods

### Information Sources, Databases, and Searching Process

The search engines of PubMed, Web of Science, and ScienceDirect were used to search the MEDLINE, Web of Science Core Collection, and ScienceDirect databases, respectively, for peer-reviewed publications that were published until February 28, 2022. The search strategy was customized for each selected database according to their filtering options (eg, for PubMed, a combination of Medical Subject Headings and other index terms was used).

The final search string included *(“mhealth” OR “telemedicine” OR “mobile application”) AND (“older adults”) AND (“activation” OR “physical activity” OR “self-healthcare” OR “healthy ageing”)*. The results that were generated by using the abovementioned search strategy in all databases were uploaded to Rayyan (Rayyan Systems Inc) [21] for the cleaning and selection process. First, the titles and abstracts of identified studies were independently screened by 2 reviewers to select relevant studies. Second, the full texts of potentially relevant studies were obtained and independently reviewed. A third assessor, who was not part of the previous screening of articles, decided on the inclusion or exclusion of the articles in cases of conflicts.

### Selection of Studies and Data Extraction

Guidelines of the PRISMA (Preferred Reporting Items for Systematic Reviews and Meta-Analyses) statement were used for the reporting of this systematic review [22]. The three databases and the reference lists of the included articles were searched and evaluated based on a set of inclusion and exclusion criteria.

Articles that met the following criteria were included: (1) a target group with an average age of ≥55 years; (2) participants without pre-existing chronic medical diseases (eg, diabetes) or mental and physical impairments (eg, repetitive falling); (3) articles that reported about digital physical activity intervention(s); (4) articles that assessed physical activity effectiveness through objective methods (eg, pedometer), subjective methods (eg, questionnaires), or a combination of objective and subjective methods; and (5) methods were embedded in one of the following designs: randomized controlled trial, quasi-experimental, clinical, or feasibility study designs.

Articles that met at least one of the following exclusion criteria were excluded: (1) articles not written in English; (2) no access to the full text; (3) a target group with an average age of <55 years; (4) a focus on older adults with pre-existing chronic medical diseases or in acute rehabilitation scenarios (eg, patients in rehabilitation after stroke); (5) articles that did not include a digital physical activity intervention; (6) articles that did not report about intervention effectiveness for physical activity; and (7) nonempirical research (eg, editorials and commentary papers).

After the selection of relevant articles, the data were extracted, and quality was assessed. The data extracted from each selected study included the author, year of publication, study design, sample size, population, intervention, technology, timescales, outcome measures, and main findings. The delivery modes of the user interaction interfaces were classified (1) as a personalized exercise introduction session at the beginning of the intervention, (2) as digital (calls, text messages, apps, the web, smartwatches, and activators), (3) as traditional (face-to-face modes and printed materials), or (4) as digital and traditional (hybrid).

The supervision of the delivery modes for the intervention groups was defined by Denton et al [23], as follows: (1) supervised (ie, physical activity is undertaken in the presence of a health care professional or qualified fitness instructor, either virtually or in person, to ensure safety and or correct technique), (2) facilitated (ie, physical activity is undertaken without the presence of a health care professional or qualified fitness instructor but with scheduled meetings or check-ins between sessions to monitor progress and provide support [virtually or in person]), or (3) unsupervised (ie, physical activity is undertaken without the presence of a health care professional or qualified fitness instructor; no support or progress tracking appointments are scheduled).

We identified and classified the used activation factors and corresponding user interfaces. The definitions of the activation factors and their classification are described in Table 1. The classification items were based on the BCT taxonomy with 16 categories developed by Michie and colleagues [11]. Additionally, the two categories suggested by Dugas and colleagues [12]—personalization and gamification—were incorporated as categories 17 and 18.

**Table 1.** Activation factors and behavior change technique taxonomy.

Activation factor		
	Skills (ability)	Knowledge (awareness)	Motivation (triggers)
Definition	A person will be equipped with skills via instructions or tips on the correct performance of the behavior. Observing others succeeding in performing the targeted activity is another strategy [24]. With increased ability, self-confidence will be fostered [9].	A person will be educated by providing them with knowledge on the benefits of sufficient physical activity as well as the consequences of insufficient physical activity [25]. The awareness that it is necessary to be active will become present, and intentions will be formed [9].	A person will be triggered to have high motivation [7,8] by gaining personalized values [26] through self-monitoring [25], monitoring by another person, monitoring via a technical device, and encouraging feedback [27].
Behavior change taxonomy categories^a,b^	Category 4: Shaping knowledge Category 6: Comparison of behavior Category 8: Repetition and substitution Category 13: Identity Category 15: Self-belief	Category 5: Natural consequences Category 9: Comparison of outcomes Category 11: Regulation	Category 1: Goals and planning Category 2: Feedback and monitoring Category 3: Social support Category 7: Associations Category 10: Reward and thread Category 12: Antecedents Category 14: Scheduled consequences Category 16: Covert learning Category 17: Personalization Category 18: Gamification

a Behavior change taxonomy categories 1 to 16 by Michie et al [11].

b Behavior change taxonomy categories 17 and 18 by Dugas et al [12].

### Quality Assessment

To assess the risk of bias, the Cochrane Collaboration tool Risk of Bias 2 (ROB 2), which focuses on different aspects of trial design (ie, conducting and reporting), was applied [28,29]. According to the tool, a study was classified as having a low risk of bias when it scored low on all 3 domains, a moderate risk of bias when 2 of the 3 domains were scored low, and a high risk of bias when 1 or no main domain was scored low. A meta-analysis was not feasible due to the selected studies being different with regard to the types of interventions.

## Results

### Studies Identified Through the Searching Process

**Figure 1.**
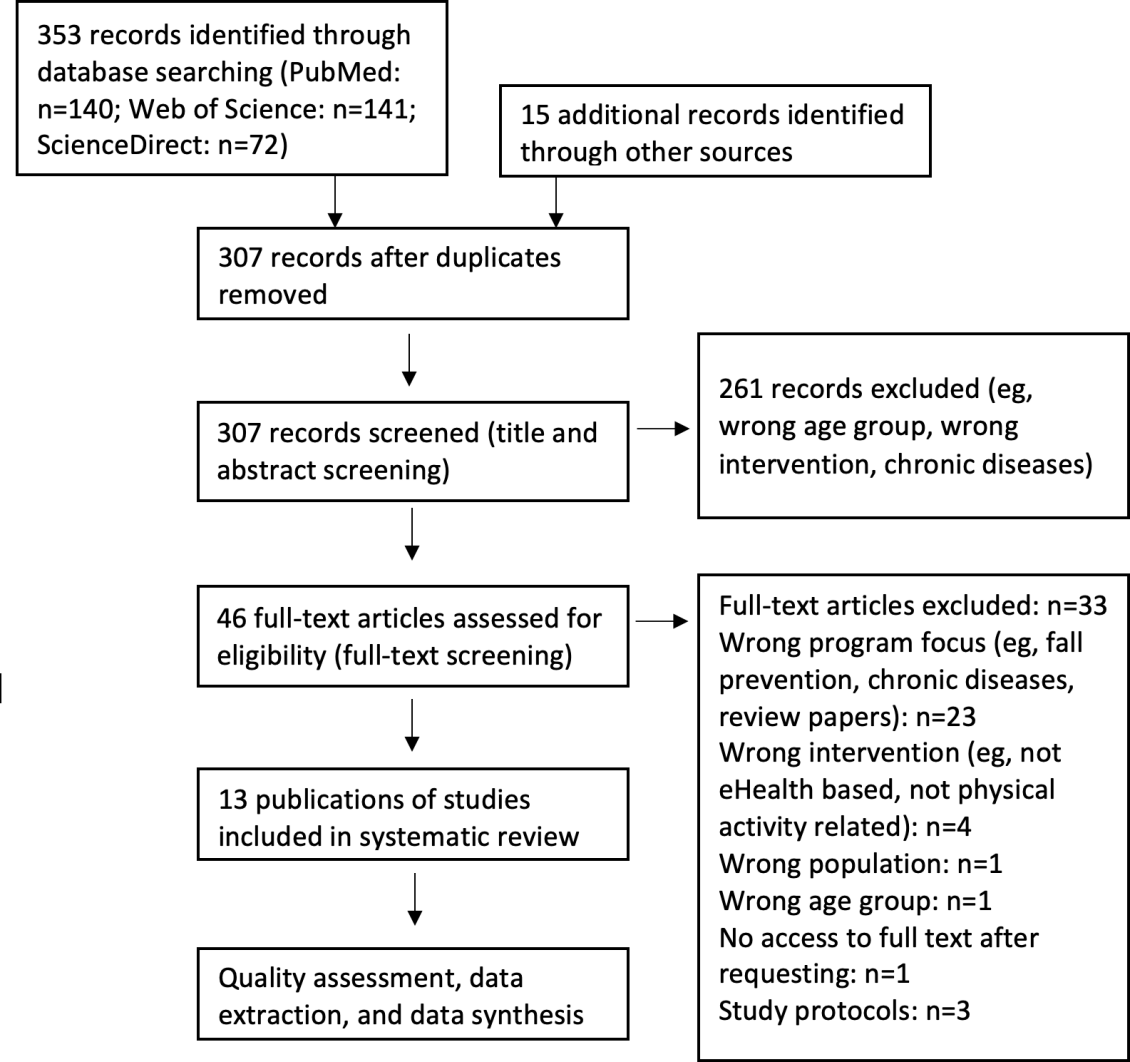
PRISMA (Preferred Reporting Items for Systematic Reviews and Meta-Analyses) diagram.

After searching the databases, 353 abstracts were identified. Further, 15 additional abstracts were identified by searching the reference lists of the initially identified articles. After the removal of duplicates and the screening of the involved abstracts, the full texts of 46 articles were assessed for eligibility. At the end, 13 articles met the inclusion criteria and were therefore included in this systematic review. Details on the study selection process are presented in Figure 1.

### Study Characteristics

#### Study Design

Of the 13 included articles, 6 were randomized controlled trials, and 8 used a quasi-experimental design. The 13 articles included a total sample of 1622 participants. Male and female participants were involved in all articles; however, their exact composition was not reported in two of them [30,31]. The mean age of the study participants ranged from 63 to 80 years. The duration of the intervention ranged from 2 weeks to 24 months. The study characteristics are presented in [30-42].

#### Physical Activity Assessment

To be able to analyze the effectiveness of an intervention, subjective or objective outcome measures for physical activity are necessary. In 4 articles (with 6 intervention groups), only subjective methods were used to measure physical activity (ie, questionnaires like the International Physical Activity Questionnaire). In 4 of the intervention groups, a significant beneficial effect of the digital or hybrid intervention was reported when compared to a control group [30-33]. In 8 articles (with 9 intervention groups), physical activity was measured by using only an objective assessment (ie, pedometer and accelerometer). In 3 of these intervention groups, a significant positive effect of the digital or traditional intervention on physical activity values was reported [31,34,35]. In the remaining intervention group, both objective assessments and subjective assessments were integrated, which showed significant effects on improving physical activity [36].

#### Activation Factors

**Figure 2.**
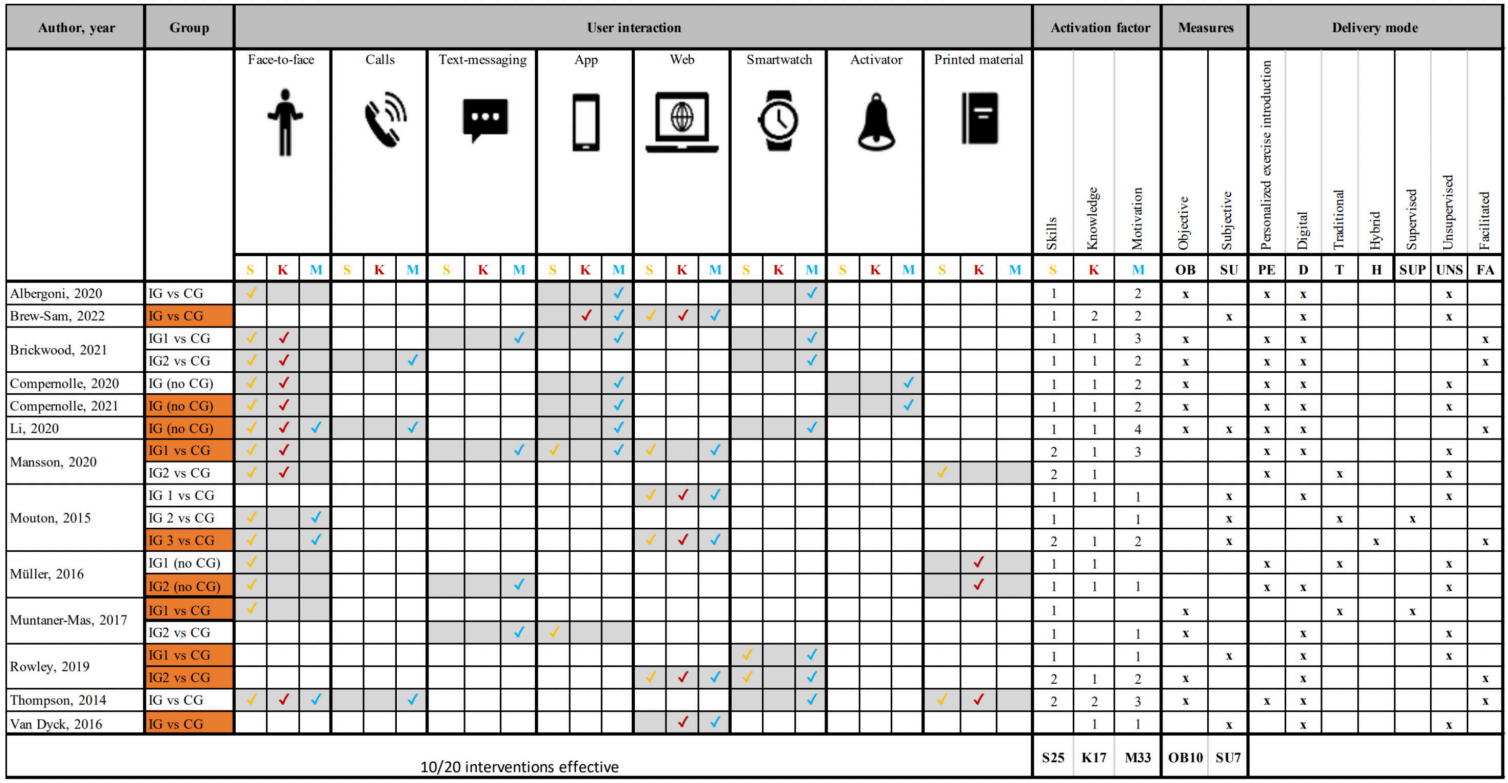
User interaction interfaces and delivery modes for delivering activation factors [30-42]. Orange blocks show intervention groups with significant beneficial findings regarding physical activity. Grey blocks highlight the user interfaces used along with the integrated activation factors. A high resolution version of the figure can be found in Multimedia Appendix 3. D: digital; FA: facilitated; H: hybrid; K: knowledge; M: motivation; OB: objective; PE: personalized exercise introduction; S: skills; SU: subjective; SUP: supervised; T: traditional; U: unsupervised.

Figure 2 presents the integrated activation factors, as well as the corresponding interfaces and delivery modes used for the user interaction in each article and intervention group. Data on the BCTs used in the intervention were extracted for each article, and the number of BCTs for each factor of activation was determined. An overview of integrated BCTs and those that were not used is presented in .

### Delivering Activation Factors

For all of the interventions, motivation was the most promoted activation factor (33 times), followed by skills (25 times) and knowledge (17 times). All 3 activation factors were identified in 12 of 20 interventions. Interventions that included all 3 activation factors were shown to be more successful in increasing or maintaining physical activity levels (articles: 7/12, 58%) compared to interventions that limited themselves to 2 or fewer activation factors (articles: 3/8, 38%).

BCTs that promoted skills, knowledge, and motivation were addressed in 19, 15, and 17 of the 20 intervention groups, respectively. Effective outcomes regarding physical activity were reported while promoting skills in 47% (9/19) of trials, while promoting knowledge in 53% (8/15) of trials, and while promoting motivation in 53% (9/17) of trials.

To promote skills, instruction on how to optimally perform a behavior was most commonly delivered in the form of training prescriptions and exercise tips (eg, key movements and where the exercises should be felt) or through descriptions using pictures (16 intervention groups). The next most common BCT for promoting skills was face-to-face demonstration or demonstration through tutorial videos (8 intervention groups), followed by the provision of information on how to integrate exercise into daily activities (habit formation; 3 intervention groups) and practice (2 intervention groups). Lastly, participants were informed about antecedents of the behavior (1 intervention group). Of the 23 available BCTs [11,12] that target skills, the aforementioned 6 (23%) were used among all included articles.

The most used strategy for changing physical activity behavior by promoting knowledge was the integration of information about the health consequences that arise if physical activity is not sufficiently part of daily behaviors (13 intervention groups). Besides that, information from a credible source (7 intervention groups) and educative information, such as the salience of consequences (6 intervention groups) and the pros and cons of physical activity (1 intervention group), were also part of the interventions. Of the 13 available BCTs [11,12] that target knowledge, the aforementioned 4 (31%) were used among all included articles.

With regard to targeting motivation, the self-monitoring of physical activity (12 intervention groups) and personalized feedback (8 intervention groups), which was often combined with individual goal setting (7 intervention groups) and social support (8 intervention groups), were the most used BCTs. Others were prompts and cues (4 intervention groups), adjustment of intervention content to the performance (4 intervention groups), action planning (2 intervention groups) and problem-solving discussions for finding ways to overcome barriers (1 intervention group). Of the 66 available BCTs [11,12] that target motivation, 11 were used among all included articles.

Altogether, of the 102 potential BCTs [11,12], 21 (21%) were integrated in all articles.

### User Interaction Interfaces and Delivery Modes

Various digital user interaction interfaces were used. The delivery modes of the user interaction interfaces varied, including personalized exercise introduction sessions at the beginning of the intervention, digital modes, traditional modes, and hybrids of digital and traditional modes. Further, 2 interventions were supervised, 12 were unsupervised, and 6 were facilitated.

To address skills, face-to-face interaction was commonly preferred (14 intervention groups). Other interaction interfaces included the web (5 intervention groups), smartwatches (2 intervention groups), apps (2 intervention groups), and printed materials (2 intervention groups). To cover knowledge, face-to-face interaction was often used (8 intervention groups), followed by the web (5 intervention groups), printed materials (3 intervention groups), and apps (1 intervention group). Calls, text messages, and activators were not used to target skills or knowledge. To target motivation, all user interaction interfaces, except printed materials, were used. Apps (7 intervention groups), smartwatches (7 intervention groups), and the web (6 intervention groups) were the most commonly integrated interaction interfaces, followed by face-to-face interaction (4 intervention groups), text messages (4 intervention groups), calls (3 intervention groups), and activators (2 intervention groups). The forms of feedback therefore differed, including visual, audio, and tactile feedback. The combination of digital and traditional user interaction modes resulted in significant positive physical activity outcomes.

Altogether, of 20 intervention groups, 14 used personal interactions, and the remaining 6 used solely nonpersonal interactions. The effectiveness of the physical activity of the nonpersonal interaction groups (intervention groups: 4/6, 67% effective) differed from the results of the personal interaction groups (intervention groups: 6/14, 43% effective).

Only Mouton and Cloes [30] reported on the delivered intervention dose, that is, how many attempts were made to contact each participant, how many attempts were received, and how many attempts were acted on by the participants. During their 3-month web-based interventions, the web-based intervention group visited the website, on average, 18 (SD 14) times, and the combined intervention group visited, on average, 39 (SD 21) times; these were much lower than the intended number of visits (potentially 90 visits).

### Risk of Bias

Table 2 presents the results of the risk of bias assessment for the 13 included articles. The risk of bias for most of the included studies (11/13, 85%) was rated as *moderate*. Only 2 of the articles showed an overall high risk of bias [30,34]. In the article by Muntaner-Mas et al [34], a convenience sample method was used, which does not exactly randomize the sample, and the allocation of participants was not concealed, thereby introducing a selection bias. In the article by Mouton and Cloes [30], 28% of the participants dropped out of the study, thereby creating a bias and affecting the effectiveness of the study. In cases where the domains of the ROB 2 were not described in the included article, the risk of bias was rated as *unclear*. In 3 articles, the blinding of participants and personnel was not possible (performance bias) [30,34,37]. Furthermore, in 3 articles, the blinding of the outcome assessment was not possible [30,33,38] because subjective methods or a combination of subjective and objective methods was used to measure physical activity (detection bias). Additionally, 2 articles showed a high risk of attrition bias (incomplete outcome data) [30,31]. Other possible biases included small sample sizes [36,39], self-selection bias, baseline differences between study groups, and short intervention periods [35,36,39,40].

**Table 2.** Cochrane collaboration tool for assessing risk of bias [28,29].

Author, year (study design)	Selection bias		Detection bias		Attrition bias	Reporting bias	
	Random sequence generation	Allocation concealment	Blinding of participants and personnel	Blinding of outcome assessment	Incomplete outcome data	Selective reporting	Summary of risk of bias
Albergoni et al [39], 2020 (pilot study)	?^a^	?	+^b^	?	+	+	±^c^
Brew-Sam et al [32], 2022 (quantitative study)	?	?	?	?	+	+	±
Brickwood et al [41], 2021 (RCT^d^)	+	+	?	?	+	+	±
Compernolle et al [40], 2020 (mixed methods study)	?	+	?	?	+	+	±
Compernolle et al [35], 2021 (mixed methods study)	?	+	?	?	+	+	±
Li et al [36], 2020 (pilot feasibility study)	?	?	?	?	+	+	±
Mansson et al [42], 2020 (feasibility study)	+	?	+	?	?	+	±
Mouton and Cloes [30], 2015 (parallel group RCT)	+	+	?	–^e^	–	?	–
Müller et al [38], 2016 (2-arm RCT)	+	+	+	–	+	?	±
Muntaner-Mas et al [34], 2017 (pilot 3-group CTS^f^)	–	–	?	+	?	?	–
Rowley et al [31], 2019 (RCT)	+	+	+	?	–	+	±
Thompson et al [37], 2014 (RCT)	+	+	?	+	+	?	±
Van Dyck et al [33], 2016 (RCT)	+	+	+	–	+	?	±

a Unclear risk of bias.

b Low risk of bias.

c Moderate risk of bias.

d RCT: randomized controlled trial.

e High risk of bias.

f CTS: clinical trial study.

## Discussion

### Key Findings

This systematic review, as a primary objective, aimed to identify which of the three activation factors—skills, knowledge, and motivation—were promoted in ICT interventions for older adults and which corresponding BCTs were applied. The secondary objective was to classify the user interaction interfaces and delivery modes that promoted these activation factors. A summary of the main findings from the 13 included articles are presented in Textbox 1.

Textbox 1. **Summary of the main findings.**

**All activation factors**

- Integrating all 3 activation factors proved most effective for increasing physical activity levels

**Skills**

- Promoted in 19 of 20 interventions
- Six BCTs were used; main behavior change techniques (BCTs) were the instruction of optimal behavior performance and the demonstration of behavior

**Knowledge**

- Promoted in 15 of 20 interventions
- Four BCTs were used; main BCTs were providing information about health consequences, information from credible sources, and information about the salience of consequences

**Motivation**

- Promoted in 17 of 20 interventions
- Eleven BCTs were used; main BCTs were self-monitoring, feedback on behavior, social support, and goal setting

**User interaction interfaces**

- A mixture of interfaces raised the chances of addressing all activation factors, which resulted in effective interventions

**Digital delivery modes**

- Commonly used to target motivation
- Allowed for the automatic detection of activity patterns and personalized feedback

**Traditional delivery modes**

- Commonly used to target skills and knowledge
- Only successful if they supported digital user interaction interfaces

### Subjective and Objective Outcome Measures

In this review, articles with subjective measures reported more positive outcomes than those using objective measures alone. In agreement with literature, articles that used only subjective methods to measure physical activity showed a positive outcome of the intervention, including improved physical activity levels [29]. This however could have possibly been due to self-reporting bias, whereby participants assess their own improvement over the intervention period [43,44]. Nevertheless, in the article that used a combination of subjective and objective factors, the authors also reported that their intervention was effective in improving or maintaining physical activity levels [36].

### Delivering Activation Factors

Although a large variety of BCTs were integrated to promote activation factors, our findings show that for each activation factor, only a limited number of BCTs from Michie et al [11] and Dugas et al [12] were exploited. Preissner et al [45] highlighted the relevance of multiple behavioral determinants (ie, social cognitive, habitual, automatic, postintentional, and planning processes) to physical activity intention in older adults. Additionally, adult users themselves have identified a variety of BCTs to fulfill their needs and preferences for engagement with physical activity apps [46]. Our findings coincide with previous reports and indicate that BCTs that integrated a combination of all 3 activation factors indeed showed the most beneficial physical activity effects. This was also reported in a review that analyzed internet-based interventions promoting health behavioral change [47].

Improving skills results in better outcomes of interventions [48]. The most used BCTs for promoting this activation factor in the interventions of the selected studies were instructions on how to perform a behavior and demonstration of a behavior, which have proven effective in improving physical activity levels [49,50]. The integration of some other likely effective BCTs was not reported, or these BCTs were not used (eg, behavioral experiments, habit reversal, graded tasks, or the identification of self as a role model). In other studies, habit reversal [51,52] and graded tasks [50] were shown to be effective in reducing sedentary behavior and improving physical activity. These can as well be integrated into interventions using ICT tools, to increase the chance of effectiveness.

To increase knowledge, 4 BCTs that focused on information about health consequences, information from credible sources, and information about the salience of consequences were integrated. In the literature, providing information about health consequences is likewise often used to target the user’s knowledge and has proved effective in reducing sedentary behavior and improving physical activity [49,53]. Other strategies (eg, providing information about social, environmental, or emotional consequences) are also available and can improve effectiveness, as seen in a systematic review with a meta-analysis [54].

To target participants’ motivation, 11 BCTs, such as self-monitoring, feedback on behavior, social support, adjusting intervention content to the performance and goal setting, were used. Gamification was not used to motivate participants, even though studies show that it can also be effective as a mode of motivation [12]. Prompts and cues were used in some interventions, but considering their effectiveness in mobile health apps [12], they should be incorporated more frequently in future interventions. Other BCTs, such as self-rewards [50] and reduced rewards [55], were also not used.

### User Interfaces and Delivery Modes

Of the 20 interventions, most (n=14, 70%) still used a personal interaction. Of course, personal interaction enables the addressing of users’ issues and gaps in skills and knowledge (eg, raising questions and receiving a demonstration of the physical activity behavior). However, based on our findings, interventions with nonpersonal interaction can be just as effective as or even more effective than interventions with personal interactions in promoting physical activity among the population of older adults. Personal interactions are time and cost intensive, and our findings suggest that solely integrating nonpersonal interactions into physical activity interventions could be a good alternative option. However, a combination of various interfaces increases the chance of addressing all activation factors, as shown by op den Akker et al [56]. Evita—a mobile, 3D virtual fitness trainer—guides users through explanations and demonstrations of how to perform exercises. An external sensor, which is attached to a belt on the patient’s hip, measures physical activity behavior. Feedback messages (eg, “you have taken more rest – take a nice walk”) are also sent to the users. The advantage of digital devices is the possibility of personalized and regular feedback [57,58]. For a hybrid intervention with a mixture of traditional (face-to-face) delivery modes targeting skills and digital (web) delivery modes targeting knowledge and motivation, positive physical activity effects were detected [30]. However, for some individuals, participating with others can still be a source of inspiration to improve or maintain physical activity levels [59].

### Issues With Digital Interfaces

We also reported digital interventions without significant beneficial effects on physical activity. Our results indicate that interventions involving the unsupervised usage of digital technology may be challenging for participants because of, for example, problems with log-ins, as well as access to or an abundance of information regarding physical activity (eg, recommendations, success stories, tips for exercise, goal setting, physical activity diaries, tools to measure physical activity, and local physical activity opportunities) [30]. A focus group with 46 older adults affirmed this argument; one of the main results of the focus group was that a web-based physical activity program was preferred to be simple; be not cluttered; and include personalized advice, reminders to check-in, and the ability to review goals [60]. An initial instruction and a helpline are useful for necessary technological support [61].

### Limitations and Recommendations

The database search was restricted to studies published only in English. Additionally, a quantitative data synthesis (ie, meta-analysis) was not feasible because the included studies were too dissimilar in terms of the intervention content, duration, assessment of outcome measures, follow-up, and comparator groups. Future articles should consider a standard method of assessing outcome measures (possibly a combination of both subjective methods and objective methods), such as using trackers for activity levels, the Behavioral Regulation in Exercise Questionnaire for covering knowledge and motivation levels [62], and the eHealth Usability Benchmarking Instrument for measuring usability (retention and acceptability) [63]. These would make the results more homogeneous for meta-analyses. Further, using the ROB 2 tool was difficult because some of its criteria could not be accurately applied to public health interventions (eg, blinding of study personnel or participants), or the information needed to determine the categories for risk of bias was not provided or was unclear in the publication. Lastly, during our research, we noticed that some studies did not report or sufficiently describe which BCTs were included.

### Conclusions

Motivation was the most promoted activation factor in the ICT interventions. However, integrating BCTs that promote all activation factors resulted in better effects in improving physical activity compared to the effects of using only 1 or 2 activation factors. Although a broad variety of BCTs were used in the articles, they were limited to about 21% (21/102) of available BCTs. Hence, many more BCTs could be exploited in future interventions. Integrating multiple interaction interfaces (eg, interfaces for delivering the intervention program and tracking one’s own activity to guarantee regular and personalized feedback) was shown to be the most effective in promoting physical activity. Time-consuming and costly personal interactions are not crucial for increasing physical activity in the older population, though they are effective in supporting digital interactions. At present, study outcomes are too diverse, which hinders intervention comparisons. To make the effects of interventions comparable, future studies should report both objective measures and subjective measures.

## Supplementary material

10.2196/42968 Multimedia Appendix 1 Study characteristics.

10.2196/42968 Multimedia Appendix 2 Behavior change technique overview.

10.2196/42968 Multimedia Appendix 3 High resolution version of Figure 2. User interaction interfaces and delivery modes for delivering activation factors [30-42] Orange blocks show intervention groups withsignificant beneficial findings regarding physical activity. Grey blocks highlight the user interfaces used along with the integrated activation factors. D: digital; FA: facilitated; H: hybrid; K: knowledge; M: motivation; OB: objective; PE: personalized exercise introduction; S: skills; SU: subjective; SUP: supervised; T: traditional; U: unsupervised.

10.2196/42968 Checklist 1 PRISMA (Preferred Reporting Items for Systematic Reviews and Meta-Analyses) checklist.

## Abbreviations

BCT: behavior change technique
ICT: information and communication technology
PRISMA: Preferred Reporting Items for Systematic Reviews and Meta-Analyses
ROB 2: Risk of Bias 2
