# Correction: The Importance of Activating Factors in Physical Activity Interventions for Older Adults Using Information and Communication Technologies: Systematic Review

**DOI:** 10.2196/55486

**Published:** 2024-02-01

**Authors:** Ellen Bentlage, John Jnr Nyamadi, Rosemary Dubbeldam

**Affiliations:** 1 Department of Movement Science, Institute of Sport and Exercise Sciences, University of Münster, Münster, Germany

 In “The Importance of Activating Factors in Physical Activity Interventions for Older Adults Using Information and Communication Technologies: Systematic Review” (JMIR Mhealth Uhealth 2023; 11(1): e42968) the authors noted some errors].

In the “Conclusions” section of the Abstract, the following phrase:

*So far, only a limited number of available BCTs (21/99, 21%) have been integrated*.

Has been changed to:

*So far, only a limited number of available BCTs (21/102, 21%) have been integrated*.

In the “Introduction” subsection “Activation Using Information and Communication Technology,” the following sentence:

*Dugas and colleagues [12] added 2 more categories (ie, gamification and personalization*).

Has been changed to:

*Dugas and colleagues [12] added 2 more categories (ie, personalization and gamification), including 9 BCTs in total*.

In the “Methods” subsection “Selection of Studies and Data Extraction,” the line:

*Additionally, the two categories suggested by Dugas and colleagues [12]—gamification and personalization—were incorporated as categories 17 and 18*.

Has been changed to:

*Additionally, the two categories suggested by Dugas and colleagues [12]—personalization and gamification—were incorporated as categories 17 and 18*.

In Table 1, under the “Skills (ability)” column, the following text:

- *Category 7: Repetition and substitution*

Has been changed to:

- *Category 8: Repetition and substitution*

Elsewhere in Table 1, under the “Knowledge (awareness)” column, the following content:

*Category 9: Comparison of outcomes*

*Category 11: Regulation*

*Category 14: Scheduled consequences*

*Category 16: Covert learning*

Has been changed to:

- *Category 5: Natural consequences*
- *Category 9: Comparison of outcomes*
- *Category 11: Regulation*

In Table 1, under the “Motivation (triggers)” column, the following text:

- *Category 1: Goals and planning*
- *Category 2: Feedback and monitoring*
- *Category 3: Social support*
- *Category 8: Associations*
- *Category 10: Reward and thread*
- *Category 12: Antecedents*
- *Category 17: Gamefication*
- *Category 18: Personalization*

Has been changed to:

- *Category 1: Goals and planning*
- *Category 2: Feedback and monitoring*
- *Category 3: Social support*
- *Category 7: Associations*
- *Category 10: Reward and thread*
- *Category 12: Antecedents*
- *Category 14: Scheduled consequences*
- *Category 16: Covert learning*
- *Category 17: Personalization*
- *Category 18: Gamification*

In the “Results” subsection “Delivering Activation Factors”, the following sentence:

*Of the 22 available BCTs [11,12] that target skills, the aforementioned 5 (23%) were used among all included articles*.

Has been changed to:

*Of the 23 available BCTs [11,12] that target skills, the aforementioned 6 (23%) were used among all included articles*.

Further in the same section, the following paragraphs:

*Others were prompts and cues (4 intervention groups), social comparison (1 intervention group), and problem-solving discussions for finding ways to overcome barriers (1 intervention group). Of the 64 available BCTs [11,12] that target motivation, 12 (19%; inclusive of the 7 aforementioned BCTs) were used among all included articles*.

*Altogether, of the 99 potential BCTs [11,12], 21 (21%) were integrated in all articles*.

Have been changed to:

*Others were prompts and cues (4 intervention groups), adjustment of intervention content to the performance (4 intervention groups), action planning (2 intervention groups) and problem-solving discussions for finding ways to overcome barriers (1 intervention group). Of the 66 available BCTs [11,12] that target motivation, 11 were used among all included articles*.

*Altogether, of the 102 potential BCTs [11,12], 21 (21%) were integrated in all articles*.

Within Textbox 1, the “Skills” subheading which previously appeared as:

*Skills*

- *Promoted in 19 of 20 interventions*
- *Five BCTs were used; main behavior change techniques (BCTs) were the instruction of optimal behavior performance and the demonstration of behavior*

Has been changed to:

*Skills*

- *Promoted in 19 of 20 interventions*
- *Six BCTs were used; main behavior change techniques (BCTs) were the instruction of optimal behavior performance and the demonstration of behavior*

Further within Textbox 1, the “Motivation” subheading previously read as:

*Motivation*

- *Promoted in 17 of 20 interventions*
- *Twelve BCTs were used; main BCTs were self-monitoring, feedback on behavior, social support, and goal setting*

And will now appear as:

*Motivation*

- *Promoted in 17 of 20 interventions*
- *Eleven BCTs were used; main BCTs were self-monitoring, feedback on behavior, social support, and goal setting*

Within the “Discussion” subsection “Delivering Activation Factors,” the sentence:

*To target participants’ motivation, 12 BCTs, such as self-monitoring, feedback on behavior, social support, and goal setting, were used*.

Was changed to:

*To target participants’ motivation, 11 BCTs, such as self-monitoring, feedback on behavior, social support, adjusting intervention content to the performance and goal setting, were used*.

The “Discussion” subsection “Conclusions” includes the claim that:

*Although a broad variety of BCTs were used in the articles, they were limited to about 21% (21/99) of available BCTs*.

This has been adjusted to appear as:

*Although a broad variety of BCTs were used in the articles, they were limited to about 21% (21/102) of available BCTs*.

In Multimedia Appendix 1, the rows “Li, 2020,” “Mansson, 2020,” “Rowley, 2019,” and “Van Dyck, 2016” have been amended from:

*18 Personalization*

And now appear as:

*17.4 Adjusting intervention content to performance*

Finally, Multimedia Appendix 2 has been changed to match the main manuscript in the ways listed below.

The “Skills” column has been changed from:

*Skills (30 BCTs*)

*5 BCTs*

To read as:

*Skills (23 BCTs*)

*6 BCTs*

The “Knowledge” column has been changed from:

*Knowledge (27 BCTs*)

*4 BCTs*

To read as:

*Knowledge (13 BCTs*)

*4 BCTs*

The “Motivation” column has been changed from:

*Motivation (50 BCTs*)

*12 BCTs*

To appear as:

*Motivation (66 BCTs*)

*11 BCTs*

The correction will appear in the online version of the paper on the JMIR Publications website on February 1, 2024, together with the publication of this correction notice. Because this was made after submission to PubMed, PubMed Central, and other full-text repositories, the corrected article has also been resubmitted to those repositories.

## Supplementary material

10.2196/55486 Multimedia Appendix 1 Study characteristics.

10.2196/55486 Multimedia Appendix 2 Behaviour change techniques overview corrected.

