# Supplementary material for: The Importance of Activating Factors in Physical Activity Interventions for Older Adults Using Information and Communication Technologies: Systematic Review
Source: JMIR Mhealth Uhealth. 2023 Oct 24;11:e42968. doi: 10.2196/42968 (PMC10644949; doi:10.2196/42968)
Supplement: Multimedia Appendix 1 [file mhealth-v11-e42968-s001.docx]

This is a Multimedia Appendix to a full manuscript published in the JMIR mHealth and uHealth (JMU). For full copyright and citation information see https://doi.org/10.2196/42968

Multimedia Appendix 1: Study characteristics ^a-n^

| Author, year | sample size (n), age, sex, Groups, user interaction interfaces | Intervention including activating factors  (S, K, M) | S | K | M | Outcome measurements related to physical activity and time points measured | Intervention effects related to physical activity | Conclusion |
| --- | --- | --- | --- | --- | --- | --- | --- | --- |
| Albergoni, 2020 | n=10,  mean age=73.8, 6 males  IG, no CG  F2f, app, smartwatch | Participants were introduced in an introduction session to a walking programme to target 50/80% of heart rate reserve via smartwatch. Weekly programme consisted of 30 min/ day on 5 days per week on walking and 25 min/day on 3 days per week exercising. Instruction to perform walk in bouts of minimum 10 minutes **[S].**  App showed whether the daily walking minutes and exercise targets had been reached **[M].** | 4.1 Instruction on how to perform the behaviour  8.3 Habit formation |  | 1.1 Goal setting  2.3 Self-monitoring of behaviour | Walking minutes and heart rate exercise target.  Baseline week, two weeks of intervention, post | Walking and exercise adherence did not increase  during the intervention  (p = 0.38, p = 0.65). | Monitoring and interface technologies can be useful to promote adherence to PA. However, personalisation must be included in the final offer. |
| Brew-Sam,  2022 | n=100 mean age=71,28,  23 males  IG, CG  IG: App, web | IG: Exercise tutorials via the website, exercise videos via YouTube **[S].**  Information on fall-prevention via website. Notifications were sent through app (e.g., walking is not enough; doing strength, balance and flexibility exercises is more effective to prevent falls, do simple strength, balance, and flexibility exercises regularly at least 20 minutes, two times a week **[K].**  M: Exercise challenges via Facebook page and app, testimonial broadcast via YouTube **[M].**  CG: No campaign interaction. | 4.1 Instruction on how to perform the behaviour  6.1 Demonstration of the behaviour | 5.1 Information about health consequences  9.1 Credible source | 6.2 Social comparison  2.3 Self-monitoring of behaviour | Frequency of exercise, strength, balance & flexibility exercises using the two questions:   1. In the last two months, how often did you exercise?  2. In the last two months, how often did you do strength, balance & flexibility exercises?  Baseline, 10-week intervention, post | A significant increase in strength, balance and flexibility exercises were reported (p<0.05). | Overall, the evaluation of the campaign impact illustrated how an integration of digital and mobile channels for communicating supportive messages, increasing awareness effectively provide means to reach older adults to encourage strength, balance and flexibility exercises to reduce their risk of falling. |
| Brickwood, 2021 | n=117, mean age=72.4,  42 males  IG1, IG2, CG  IG1: F2f, smartwatch, app with text-messages  IG2: F2f, smartwatch, calls | IG 1: 12-week exercise **[S]** and education **[K]** programme led by an exercise physiologist. Then activity tracking via smartwatch, daily text message feedback via app based on smartwatch steps **[M],** f2f discussion for identifying and addressing barriers to be active with exercise tips from an AEP **[S].**  IG 2: 12-week exercise **[S]** and education **[K]** programme led by an exercise physiologist. Then activity tracking via smartwatch, fortnightly calls for the first three months, then monthly calls until end of the intervention regarding well-being check, feedback based on steps relative to goals, positive reinforcement **[M]**, barriers to walking, problem solving discussion, exercise tips **[S]**.  CG: Individualised home-based exercise programme. | 4.1 Instruction on how to perform the behaviour  4.1 Instruction on how to perform the behaviour | 5.1 Information about health consequences  9.1 Credible source  5.1 Information about health consequences  9.1 Credible source | 2.3 Self-monitoring of behaviour  2.2 Feedback on behaviour  2.3 Self-monitoring of behaviour  2.2 Feedback on behaviour  3.1 Social support (unspecified) | Average daily step count via smartwatch  before the end of intervention at 3, 6 and 12 months. Only days with complete recordings were considered and daily step counts were averaged for each assessment time point.  Baseline,  after 3, 6 and 12 months | During the 12-month period, both IGs maintained daily steps, whereas the CG showed a reduction in daily steps.  IG1: MD −588 steps, 95% CI −1359 to 182 steps; P=0.09  IG2: MD −79 steps, 95% CI −823 to 663 steps; P=0.81  CG: MD 981 steps, 95% CI −1668 to −294 steps; P=0.003 | Both interventions are effective at maintaining PA levels in older adults following a structured lifestyle intervention. |
| Compernolle, 2020 | n=28, mean age=64.3, 13 males  IG, no CG  F2f, activator, app | Participants received a presentation by an expert on SB **[K][S].**  Then a self-monitoring-based mhealth intervention was introduced. The activator is worn on the front of the thigh and provides visual and tactile feedback.  Visual feedback is constantly available and can be viewed continuously via the app. Tactile feedback is provided by means of a vibration of the activator device itself each time a participant is sitting for 30 uninterrupted minutes. If a participant remains sedentary, the vibration is repeated after another 30 minutes. Participants were able to turn the vibration on and off **[M].** | 6.1 Demonstration of the behaviour | 5.1 Information about health consequences  9.1 Credible source | 2.3 Self-monitoring of behaviour  2.2 Feedback on behaviour  7.1 Prompts/cues | Total sedentary time, sit-to-stand transitions, standing time, and number of steps were objectively estimated by means of the activPAL accelerometer.  1 week baseline, 3 intervention weeks, 1 week post | Sitting and standing time were very similar at baseline and post-measurements. Small improvement in steps of around 400 per day (not significant). | The intervention made them more aware of their SB, but the intervention did not result in a decrease in sedentary time. |
| Compernolle, 2021 | n=26 mean age= 64.4,  13 males  IG, no CG  F2f, activator, app | IG: Participants received a presentation by an expert on SB **[K][S]** then a self-monitoring-based mhealth intervention was introduced. The activator is worn on the front of the thigh and provides visual and tactile feedback.  Visual feedback is constantly available and can be viewed continuously via the app. Tactile feedback is provided by means of a vibration of the activator device itself each time a participant is sitting for 30 uninterrupted minutes. If a participant remains sedentary, the vibration is repeated after another 30 minutes. Participants were able to turn the vibration on and off **[M].**  No CG. | 6.1 Demonstration of the behaviour | 5.1 Information about health consequences  9.1 Credible source | 2.3 Self-monitoring of behaviour  2.2 Feedback on behaviour  7.1 Prompts/cues | Activator device collected data to determine the wearer’s body posture, like sitting, lying, upright and stepping speed. Accelerometer data was used to estimate the proximal outcome, which was a break in SB within 1, 3, and 5 minutes after receiving personalized haptic feedback. Time-related characteristics of when the haptic feedback was provided were extracted from the system usage data of the activator and categorized into the following six categories: 6-9 AM,  9 AM-noon, noon-3 PM, 3-6 PM, 6-9 PM,  9 PM-midnight.  3-week intervention, no baseline, or post-measurements | 2628 vibrations were provided to the users. Of these, 379 (14.4%), 570 (21.7%) and 798 (30.4%) resulted in a SB break within 1, 3 and 5 minutes,  respectively. Although the 1-minute interval did not reveal significant differences in the percentage of breaks depending on the time at which the haptic feedback was provided, the 3- and 5-minute intervals did show significant differences in the percentage of breaks depending on the time at which the haptic feedback was provided. Concretely, the percentage of SB breaks was significantly higher if personalised haptic feedback was provided between noon and 3 PM compared to if the feedback  was provided between 6 and 9 AM (odds ratio 1.58, 95% CI 1.01-2.47, within 3 minutes: odds ratio 1.78, 95% CI 1.11-2.84,  within 5 minutes). | Most haptic vibrations, especially those received in the morning, did not result in a break in SB. Older adults were more likely to break their SB in the afternoon. Simply bringing habitual SB into conscious awareness seems to be insufficient to target SB. More research is needed to optimize push components in interventions aimed at the reduction of SB in older adults. |
| Li, 2020 | n=8, mean age=74,  2 males  IG, no CG  F2f, calls, app, smartwatch | IG:  2-hour personalised f2f PA education session with an exercise trainer including:  General rules of PA for older adults **[K],** PA prescription with training consisting of steps, strength, flexibility, and balance activities **[S],**  self-monitoring of PA via smartwatch, app which sends sedentary alerts  to encourage elderlies to reach their daily goals. Google calendar app to send reminders for scheduled activity, daily step goals and motivation messages. Weekly phone consultation with trainer to receive guidance and adjustment on activity plans, and weekly financial incentives **[M].** | 4.1 Instructions on how to perform the behaviour | 5.1 Information about health consequences  9.1 Credible source | 2.3 Self-monitoring of behaviour  7.1 Prompts/cues  3.1 Social support (unspecified)  1.1 Goal setting  17.4 Adjusting intervention content to performance  10.1 Material incentive | PA was assessed using Actiwatch 2 worn on the non-dominant wrist to provide an objective estimate of the 24-hour physical movement. Different variables were extracted:  1) Mean level of PA during the daytime (counts/minute).  2) Mean duration of SB during the daytime.  3) Mean percent of daytime that the participant spent on SB.  PASE at the baseline and post-intervention visits.  1 week pre-test, 4-week intervention, 1 week post-test | 1) On average, the level of daytime PA increased significantly at post-test (D=41.5 counts/minutes, 95% CI=[8.8, 74.4], P=0.02).  2) Compared with baseline, the sedentary time decreased significantly during the intervention (D [mean difference] =42.3 min, 95%CI= [79.4,5.2], P=0.03) and at post-intervention (D=87.4 min, 95%CI=[133.5,30.4], P=<0.01).  3) The participants spent less of their waking time on SB both during the intervention (D=5.7%, 95%CI=[10.1,1.3], P==0.019) and at post-test (D=8.0%, 95%CI=[12.1,3.6], P=<0.01).  PASE score increased at the post-test (D=96.2, 95% CI=[15.8,176.5], P=0.025). | This pilot intervention provides insight into a potentially novel approach involving mhealth technology for older adults to achieve and maintain active lives. |
| Mansson, 2020 | n=67, mean age=77,  19 males  IG1, IG2  IG1: F2f, text-messaging, app, web  IG2: F2f, printed material, | IG1+2: All participants attended an introduction meeting with education about accidental falls and fall prevention and the introduction to the exercise programme **[K].**  The recommendations were to exercise 30 min at least three times per week **[S].**  IG1:  Digital Programme provided exercises which are instructed in short video clips that image older persons doing the exercises. Participants build their own exercise programme by selecting one exercise from each of ten predetermined groups of exercises to improve strength, balance, and gait/step ability **[S].** Participants receive support in the form of written motivational feedback from a virtual physiotherapist (computer generated pre-written messages, delivered according to the participants’ reported exercise),  exercise planning and possibility to review the exercise diary **[M].** Also, they received examples on how to integrate exercises into daily activities and practice outdoors **[S].**  IG2: Paper-based exercises with drawings and written instructions.  In order to help the participants build their programme, the exercises were divided in two sections with strength or balance exercises. Each section was further arranged into three different levels of difficulty with was a modification from the Otago Home Exercise Programme Booklet. Participants were instructed to select five exercises from each section to build a programme of ten exercises **[S].** | 4.1 Instruction on how to perform the behaviour  6.1 Demonstration of the behaviour  8.3 Habit formation  4.1 Instruction on how to perform the behaviour  6.1 Demonstration of the behaviour | 5.1 Information about health consequences  5.2 Salience of consequences | 17.4 Adjusting intervention content to performance  3.1 Social support (unspecified)  2.2 Feedback on behaviour  2.3 Self-monitoring of behaviour | Activity level was measured  with the SGPALS.  Balance and and functional strength was assessed with the SPPB.  Exercise diaries were filled out either digitally (IG1) or paper based (IG2).  Four sub-groups were created to compare  adherence with the following  definitions:  1. Enrolled, everyone that started the intervention.  2. Completed study, all participants that did not  explicitly withdraw from the exercise programme,  independent of the degree of participation.  3. Exercise completion ≥75% of the weeks, participants  that self-reported exercise at least one session per  week for 12 of the 16 weeks.  4. Exercise duration ≥75%, participants that self-reported at least 75% of the recommended 90 min of exercise per week (at least in total 1080 min over  16 weeks).  Baseline, 4-month intervention, 12 month-follow-up | In both groups 50–59% reported exercise at least 75% of the intervention period. The only significant difference  for adherence was in the subgroup that completed ≥75% of exercise duration, the digital programme users exercised  more minutes per week (p= 0.001).  At 12 months follow-up 67% of participants using the digital programme continued to exercise  regularly compared with 35% for the paper booklet (p= 0.036). | Exercise interventions based on either a digital programme or a paper booklet can be used as a self-managed, independent fall prevention programme. There is a similar adherence in both programme during a 4-month  intervention, but the digital programme seems to facilitate long-term maintenance in regular exercise. |
| Mouton, 2015 | n=149, mean age=65, male= N.A.  IG1, IG2, IG3, CG  IG1: Web  IG2: F2f  IG3: Web + f2f | IG1 - Web-based intervention (PA promotion website:)  Why should I Move (i.e., benefits of PA), what is PA (i.e., the definition, the different types  and intensities of PA), recommendations for PA  **[K].**  Success stories (i.e., testimonies of older  adults who succeed in becoming physically active), tips to start (e.g., establish PA routines), fixing goals (i.e.  setting SMART objectives), overcome barriers (e.g., the weather is bad), choose an activity (regarding personality and goals), Examples of exercises (i.e., endurance, strength, balance, and flexibility exercises), local PA opportunities (i.e., PA associations and facilities in the municipality), local PA trails (i.e., cycling and walking trails in the municipality) **[S].**  PA journal (i.e., write down past activities), Tools to measure PA (i.e., pedometers and heart  rate monitors), monthly tailored automatic feedback based on answers to a questionnaire within the web **[M].**  IG2 - Center-based  Intervention:  12 sessions (one session per week) of group exercising. Program supervised by trained physical educators familiar with senior PA promotion. Each exercising session included: light  cardiorespiratory, muscular, and articular warm-up. A combination of endurance, strength, flexibility and  balance training: and a light cool-down based on  relaxation. **[S].**  On each exercising session, the physical educator gave PA motivational  advice (e.g., create a  PA calendar or fix your personal goals,) **[M].**  and PA environmental advice (e.g., be aware of the local facilities for PA in the municipality or find a PA partner in your social environment) **[S].**  IG3: Mixed (center- and web-based) intervention including access to PA website **[S, K, M]**, monthly tailored PA advice**[M]** and weekly PA group sessions **[S].**  CG: Without any intervention. | 4.1 Instruction on how to perform the behaviour  4.2 Information about antecedents  8.3 Habit formation    4.1 Instruction on how to perform the behaviour | 5.1 Information about health consequences  5.2 Salience of consequences  9.2 Pros and cons | 2.3 Self-monitoring of behaviour  2.2 Feedback on behaviour  1.1 Goal setting  1.2 Problem Solving  3.3 Social support (practical)  3.1 Social support (unspecified) | Self-reported PA data were collected using the SF-IPAQ  Baseline, 3-month intervention, 12 months after | Intervention effect compared to CG at 12 months:  IG1 Web-based  (p = 0.247, ES = 0.06)  IG2 Center-based  (p = 0.083, ES = 0.14)  IG3 Mixed  (p = 0.041, ES = 0.20):  Only the IG3 was significant effective for increasing PA level compared to the CG with a relatively small ES. For IG1 and IG2 no significant increases in PA were noticed. | Only the mixed intervention condition, comprising both online and offline components, resulted in a significant increase of PA levels at 12 months. This in- crease could mainly be attributed to the offline omponents of the intervention as the center-based (IG2) intervention was borderline effective (ES = 0.14) in increasing PA level.  Nevertheless, the addition of online components seems to be beneficial, without indicating that a web-based intervention alone is sufficient to significantly increase PA among older adults. |
| Müller, 2016 | n=43, mean age=63.28,  11 males  IG1, IG2  IG1:  F2f, printed material  IG2:  F2f, printed material, text-messages | IG1 (no text-messages):  All participants were introduced to a set of exercises in one practical session **[S]** and received an exercise booklet with included information on the benefits of exercise**[K]**, and 12 exercise descriptions **[S].**  IG2 (SMS text-messaging group):  All participants were introduced to a set of exercises in one practical session **[S]** and received an exercise booklet with included information on the benefits of exercise**[K]**, and 12 exercise descriptions **[S].**  They got additional 60 (5 weekly) text messages over 12 weeks. The content of the SMS text messages was based upon behaviour change techniques and were designed to be encouraging and act as reminders **[M].**  Participants were advised to exercise as often as possible each week to increase health benefits, but no other  formal recommendations were provided. | 4.1 Instruction on how to perform the behaviour  6.1 Demonstration of the behaviour  8.1 Behavioural practice/rehearsal    4.1 Instruction on how to perform the behaviour  6.1 Demonstration of the behaviour  8.1 Behavioural practice/rehearsal | 5.1 Information about health consequences  5.2 Salience of consequences  5.1 Information about health consequences  5.2 Salience of consequences | 7.1 Prompts/cues  3.1 Social support (unspecified)    2.3 Self-monitoring of behaviour | Weekly exercise frequency measured with an exercise  log appended to the exercise booklet. Participants were asked to record dates, times, and duration of exercise sessions.  IPAQ was used to measure frequency (days per week) and duration (hours and minutes per day) of vigorous PA, moderate PA, and walking during the previous week.  Another item asked about the time spent sitting on a normal  Weekday.  Baseline, 12-week intervention, 24-weeks after baseline | Over the 12-week intervention period, IG2 participants  exercised more frequently per week (mean 3.74, SD 1.34) compared to participants in the non-SMS texting arm  (Mean 2.52, SD 1.85). This mean difference  Was significant (t=2.30, P=0.03, d=0.76).  Weekly exercise frequency decreased by 0.43 sessions from week 12 to week 24 in the overall sample.  Significant decrease of 0.68 sessions within the SMS texting arm (F1,37=8.93, P=0.005) was analysed, whereas no significant decrease was observed in the non-SMS texting arm (F1,37 =0.85, P=0.36). | Participants who received 60 encouraging SMS text-messages over 12 weeks exercised significantly more than participants who did not receive such SMS text messages.  Exercise frequency decreased significantly in the texting arm when text-messages ceased. These findings suggest that text-messages have a strong impact on exercise participation in  older adults, but the effect does not seem to be sustainable once  they are removed. |
| Muntaner-Mas, 2017 | n=32, mean age=63.78,  8 males  IG1, IG2, CG  IG1: F2f  IG2: F2f, app, text-messages | IG1 (training group): Twice per week training sessions (warm-up, strength training, aerobic training, flexibility) on the sports ground **[S].**  IG2 (mobile group):  Twice per week training sessions (warm-up, strength training, aerobic training, flexibility) delivered WhatsApp.  All participants were added to a chat and received two videos per week with exercises which could be downloaded **[S].**  In the videos appeared a member of the research group carrying out the  prescribed exercises. Throughout multimedia material were inserted subtitles which indicated the description, intensity, and rest of each exercise **[S].** Two messages were sent to the participants per week to encourage them to be active (not tailored) **[M].**  CG: No intervention. | 4.1 Instruction on how to perform the behaviour  4.1 Instruction on how to perform the behaviour  6.1 Demonstration of the behaviour |  | 3.1 Social support (unspecified) | Muscular strength was evaluated using a handgrip dynamometer (in kg) to assess the maximal voluntary grip strength.  Static balance (s) was measured using the flamingo balance test.  Aerobic capacity (steps) was assessed using the 2-minute step test.  Baseline,  10-week intervention, post-measurement | Muscular strength:  IG1 training vs. CG:  ES = −0.51, p = 0.236  IG2 mobile vs CG:  ES = −0.04,  p = 0.937  IG1 training vs. IG2 mobile:  ES = −0.46, p = 0.337  Static balance: IG2 mobile vs. CG:  ES = −1.17,  (-2.31, 0.03) p = 0.026  IG1 training vs. CG:  ES = −0.07, (-0.89, 0.75) p = 0.867  IG1 training vs. IG2 mobile:  ES = 1.11, (0.19, 2.03) p = 0.024  Aerobic capacity: IG1 training vs. CG:  ES = −0.12, p = 0.795  IG2 mobile vs. CG:  ES = −0.73, p = 0.146  IG1 training vs. IG2 mobile:  ES = 0.61, p = 0.187 | No significant changes related to PA were identified for muscular strength and aerobic capacity in both groups.  IG1 compared to IG2 improved statistic balance significantly.  Comparison between training and mobile group showed that WhatsApp-based PA intervention was less effective than f2f condition. |
| Rowley, 2019 | n=170, mean age=66.1,  sex= N.A.  IG1, IG2, CG  IG1: Smartwatch  IG2: Web + smartwatch | IG1 (Pedometer group):  Given the goal to increase their daily step count by 10% each week until they met 10,000 steps per day**[M].** after which they were instructed to maintain 10,000 steps per day **[S].**  IG2 (Tailored internet-mediated pedometer group): Given the goal to increase their daily step count by 10% each week until they met 10,000 steps per day**[M].** after which they were instructed to maintain 10,000 steps per day **[S].**  Received the same pedometer and was instructed to log onto an interactive website weekly. The interactive website used key strategies, meaning education during weeks 1 to 3, to provide cognitive understanding of the benefits of PA, education on national recommendations **[K]** and self-awareness of current activity levels) and goal setting like increasing weekly steps by 10%, self-regulation, and frequent tailored feedback and rewarding) Uploaded steps per day were graphically represented for each day for the prior week, and plotted against nationally recommended amounts. Each week of the interactive program, users were also guided by an ongoing discussion forum, posing questions and solutions to increase PA, and access to “ask the expert” (a trained behaviourist and member of the research team) **[M].**  CG: Instructed to maintain usual PA levels. | 4.1 Instruction on how to perform the behaviour  4.1 Instruction on how to perform the behaviour | 5.1 Information about health consequences  5.2 Salience of consequences | 1.1 Goal setting  1.1 Goal setting  2.2 Feedback on behaviour  17.4 Adjusting intervention content to performance  3.1 Social support (unspecified) | IG1: Daily step counts were recorded in paper logs and mailed to researchers each week.  IG2: Daily step counts were uploaded from the pedometer at the end of each week.  Baseline, 12-week intervention, post-measurement | IG1 significantly increased average step count by 62,1%.  IG2 experienced a significant 119,4% increase.  The CG did not experience significant changes in step count from baseline to 12 weeks.  IG1 and IG2 had significantly higher step counts than the CG at 12 weeks (3,215 and 5,632).  IG2 had significantly higher steps after 12 weeks compared with IG1. | Both interventions were effective in increasing the PA.  Individually tailored internet-mediated PA interventions are an effective way to significantly  increase PA in older adults. |
| Thompson, 2014 | n=49, mean age=79.5,  9 males  IG, CG  IG: F2f, calls, smartwatch, printed materials | IG:  Wore Fitbit accelerometer for 6 months with feedback from device and exercise counselling **[M].**  Participants were f2f guided through the READY, SET, and GO steps from the Go4Life programme. Get READY provided education on general rules of PA for older adults **[K]** and on different exercises that promoted endurance, strength, balance, and flexibility **[S].**  Get SET prepared the subjects for behaviour change by setting goals and building a plan **[M].**  GO included carrying out the exercise plan developed **[S]** and tracking PA with the Fitbit**.**  The counsellor met with each subject once a week via scheduled telephone call to guide the participants. F2f feedback was provided every two months **[M].**  CG: Wore Fitbit accelerometer for 6 months without feedback from device.  After 6 months, the participants of each group changed to the other group. | 4.1 Instruction on how to perform the behaviour | 5.1 Information about health consequences  9.1 Credible source | 2.3 Self-monitoring of behaviour  1.1 Goal setting  1.4 Action planning  3.1 Social support (unspecified) | Accelerometer  was used for activity measurements.  It was worn by all subjects during weeks 1 and 2, week 14, weeks 25 and 26, week 38 and weeks 49 and 50. This accelerometer  captured accelerometer data every second and was downloaded by  the study team each week it was worn.  A second accelerometer, was worn continuously throughout the study and provided feedback.  Baseline, 6, 12 months | PA units at 6 months compared to baseline:  IG was less active (not significant)  −217.8 (±1032.3)  p = 0.31. CG was significantly less active  − 583.6,  (±940.3)  p = 0.006.  PA units at 12 months: No group or between group differences from 6 to 12 months. | Intervention was not effective in increasing PA units at 6 and 12 months. |
| Van Dyck, 2016 | n =284, mean age = 63.2, 134 males  IG, CG  IG: Web | IG:  MyPlan1.0. based on self-regulation theory and the Health Action Process Approach Model  In the pre-intentional processes based on IPAQ-values personal feedback was provided to motivate participants to change their PA **[M]** and to raise awareness. Participants were given the possibility to read more information on PA, like to the relation with health outcomes **[K]**. In the post-intentional processes participants make an action plan (what, when, where, and with whom) to bridge the gap between intentions and behaviour. They were also offered the possibility to identify difficult situations and hindering factors (i.e., coping planning). Personal action plan was sent via email with opportunity to send it to family/friends for social support (module 1). In module 2 (activated 1 week after finishing module 1) participants received feedback about behavioural change process and goals, possibility to adopt action plan. In module 3 (activated 1 month after finishing module 2) had the same content as module 2 **[M].**  CG: Only answered questionnaires. Waitlisted, received intervention after end of study. |  | 5.1 Information about health consequences  5.2 Salience of consequences | 2.2 Feedback on behaviour  2.3 Self-monitoring of behaviour  1.4 Action planning  17.4 Adjusting intervention content to performance  1.1 Goal setting  2.7 Feedback on outcome(s) of behaviour | IPAQ:  1. Total PA (min/week).  2. Moderate PA (min/ week).  3. Vigorous PA (min/week).  Baseline,  1 week intervention, 1 month follow-up | Intervention effect after 1 week:  1. Total: IG 561.2 min/week (SD 346.3), CG 623.5 min/week (SD 375.5), not significant.  2. Moderate: IG 476.7 min/week (SD 309.9), CG 515.0 min/week (SD 350.9), not significant.  3. Vigorous: IG 37.5 min/week (SD 86.7), CG 56.6 min/week (SD 115.9), not significant.  After 1 month follow-up:  1. Total: IG 663.5 min/week (SD 384.2), CG 599.7 min/week (SD 356.9), p < 0.05.  2. Moderate: IG 550.7 min/week (SD 313.7), CG 476 min/week (SD 292.3), not significant.  Vigorous PA: IG 66.3 min/week (SD 128.5), CG 63.6 min/week (SD 133.4), not significant. | Intervention effective in increasing total PA at 1-month follow-up. |

Intervention group= IG^a^, Core Group= CG^b^, Face-to-face= F2f^c^, Short Form 12 Survey= SF-12^d^, Short Questionnaire to Assess Health enhancing physical activity= SQUASCH^e^, Physical Activity= PA^f^, Sedentary Behaviour= SB^g^, mobile Health= mHealth^h^, Physical Activity Scale for the Elderly= PASE^i^ ,Saltin-Grimby Physical Activity Level Scale= SGPALS^j^, Short Physical Performance Battery= SPPB^k^, Short Form International Physical Activity Questionnaire= SF-IPAQ^l^, Not Applicable= N.A.^m^, Effect Size= ES^n^
