# Supplementary material for: The Importance of Activating Factors in Physical Activity Interventions for Older Adults Using Information and Communication Technologies: Systematic Review
Source: JMIR Mhealth Uhealth. 2023 Oct 24;11:e42968. doi: 10.2196/42968 (PMC10644949; doi:10.2196/42968)
Supplement: Multimedia Appendix 2 [file mhealth-v11-e42968-s002.docx]

This is a Multimedia Appendix to a full manuscript published in the JMIR mHealth and uHealth (JMU). For full copyright and citation information see https://doi.org/10.2196/42968

Multimedia Appendix 2: Behaviour Change Techniques Overview

| **Behaviour Change Techniques (BCTs)** | | | | | |
| --- | --- | --- | --- | --- | --- |
| **Skills (23 BCTs)** | | **Knowledge (13 BCTs)** | | **Motivation (66 BCTs)** | |
| **6 BCTs** | | **4 BCTs** | | **11 BCTs** | |
| 16 | 4.1 Instruction on how to perform the behaviour | 13 | 5.1 Information about health consequences | 12 | 2.3 Self-monitoring of behaviour |
| 8 | 6.1 Demonstration of the behaviour | 7 | 9.1 Credible source | 8 | 2.2 Feedback on behaviour |
| 3 | 8.3 Habit formation | 6 | 5.2 Salience of consequences | 8 | 3.1 Social support (unspecified) |
| 2 | 8.1 Behavioural practice/ rehearsal | 1 | 9.2 Pros and cons | 7 | 1.1 Goal setting |
| 1 | 6.2 Social comparison | 0 | 5.3 Information about social and environmental consequences | 4 | 7.1 Prompts/cues |
| 1 | 4.2 Information about antecedents | 0 | 5.4. Monitoring of emotional consequences | 4 | 17.4 Adjusting intervention content to performance (Personalisation) |
| 0 | 4.4 Behavioural experiments | 0 | 5.5. Anticipated regret | 2 | 1.4 Action planning |
| 0 | 4.3. Re-attribution | 0 | 5.6 Information about emotional consequences | 1 | 3.2 Social support (practical) |
| 0 | 6.3. Information about others' approval | 0 | 9.3. Comparative imagining of future outcomes | 1 | 2.7 Feedback on outcome(s) of behaviour |
| 0 | 8.2. Behaviour substitution | 0 | 11.1. Pharmacological support | 1 | 10.1 Material incentive |
| 0 | 8.4 Habit reversal | 0 | 11.2. Reduce negative emotions | 1 | 1.2 Problem solving |
| 0 | 8.5. Overcorrection | 0 | 11.3. Conserving mental resources | 0 | 1.3. Goal setting (outcome) |
| 0 | 8.6 Generalisation of target behaviour | 0 | 11.4. Paradoxical instructions | 0 | 1.5. Review behaviour goal(s) |
| 0 | 8.7. Graded tasks |  |  | 0 | 1.6. Discrepancy between current behaviour and goal |
| 0 | 13.1 Identification of self as role model |  |  | 0 | 1.7. Review outcome goal(s) |
| 0 | 13.2. Framing/reframing |  |  | 0 | 1.8. Behavioural contract |
| 0 | 13.3. Incompatible beliefs |  |  | 0 | 1.9. Commitment |
| 0 | 13.4. Valued self-identify |  |  | 0 | 2.1. Monitoring of behaviour by others without feedback |
| 0 | 13.5. Identity associated with changed behaviour |  |  | 0 | 2.4. Self-monitoring of outcome(s) of behaviour |
| 0 | 15.1. Verbal persuasion about capability |  |  | 0 | 2.5. Monitoring of outcome(s) of behaviour without feedback |
| 0 | 15.2. Mental rehearsal of successful performance |  |  | 0 | 2.6. Biofeedback |
| 0 | 15.3. Focus on past success |  |  | 0 | 3.3. Social support (emotional) |
| 0 | 15.4. Self-talk |  |  | 0 | 7.2. Cue signalling reward |
|  |  |  |  | 0 | 7.3. Reduce prompts/cues |
|  |  |  |  | 0 | 7.4. Remove access to the reward |
|  |  |  |  | 0 | 7.5. Remove aversive stimulus |
|  |  |  |  | 0 | 7.6. Satiation |
|  |  |  |  | 0 | 7.7. Exposure |
|  |  |  |  | 0 | 7.8. Associative learning |
|  |  |  |  | 0 | 10.2. Material reward (behaviour) |
|  |  |  |  | 0 | 10.3. Non-specific reward |
|  |  |  |  | 0 | 10.4. Social reward |
|  |  |  |  | 0 | 10.5. Social incentive |
|  |  |  |  | 0 | 10.6. Non-specific incentive |
|  |  |  |  | 0 | 10.7. Self-incentive |
|  |  |  |  | 0 | 10.8. Incentive (outcome) |
|  |  |  |  | 0 | 10.9 Self reward |
|  |  |  |  | 0 | 10.10. Reward (outcome) |
|  |  |  |  | 0 | 10.11. Future punishment |
|  |  |  |  | 0 | 12.1. Restructuring the physical environment |
|  |  |  |  | 0 | 12.2. Restructuring the social environment |
|  |  |  |  | 0 | 12.3. Avoidance/reducing exposure to cues for the behaviour |
|  |  |  |  | 0 | 12.4. Distraction |
|  |  |  |  | 0 | 12.5. Adding objects to the environment |
|  |  |  |  | 0 | 12.6. Body changes |
|  |  |  |  | 0 | 14.1. Behaviour cost |
|  |  |  |  | 0 | 14.2. Punishment |
|  |  |  |  | 0 | 14.3. Remove reward |
|  |  |  |  | 0 | 14.4. Reward approximation |
|  |  |  |  | 0 | 14.5. Rewarding completion |
|  |  |  |  | 0 | 14.6. Situation-specific reward |
|  |  |  |  | 0 | 14.7. Reward incompatible behaviour |
|  |  |  |  | 0 | 14.8. Reward alternative behaviour |
|  |  |  |  | 0 | 14.9. Reduce reward frequency |
|  |  |  |  | 0 | 14.10. Remove punishment |
|  |  |  |  | 0 | 16.1. Imaginary punishment |
|  |  |  |  | 0 | 16.2. Imaginary reward |
|  |  |  |  | 0 | 16.3. Vicarious consequences |
|  |  |  |  | 0 | 17.1 Tailoring to demographic characteristics |
|  |  |  |  | 0 | 17.2 Tailoring to health status |
|  |  |  |  | 0 | 17.3 Tailoring to psychological characteristics |
|  |  |  |  | 0 | 17.5 General/ not enough detail |
|  |  |  |  | 0 | 18.1 Earn points |
|  |  |  |  | 0 | 18.2 Earn badges/ levels |
|  |  |  |  | 0 | 18.3 Leaderboards |
|  |  |  |  | 0 | 18.4 Competitions |
